# Optimal dietary standardized ileal digestible lysine level for pigs during the grower, early and late finisher periods

**DOI:** 10.1186/s12917-022-03557-1

**Published:** 2022-12-23

**Authors:** Wenxin Song, Zijuan Wu, Wenli Li, Yali Li, Huansheng Yang

**Affiliations:** Hunan Provincial Key Laboratory of Animal Intestinal Function and Regulation, College of Life Sciences, Hunan Normal University, No.36 Lushan Road, Changsha, 410081 Hunan China

**Keywords:** Growing-finishing pig, Growth performance, Lysine requirements, Standardized ileal digestible

## Abstract

**Background:**

Lysine (Lys) is the first limiting amino acid for pigs fed corn-soybean meal diets. Three experiments were conducted to estimate the optimal standardized ileal digestible (SID) Lys requirement for growing (Exp. 1), early finishing (Exp. 2), and late finishing (Exp. 3) pigs under commercial conditions.

**Results and conclusions:**

In Exp. 1, a total of 650 growing pigs (32.21 ± 0.33 kg bodyweight), were allocated to 5 dietary treatments supplemented with 0.75, 0.85, 0.94, 1.03, and 1.13% SID Lys. Each treatment had 5 replicate pens with 26 pigs per pen. The lowest feed to gain ratio (F:G) was obtained by pigs fed the 1.03% Lys diet and F:G showed both a linear and a quadratic response with increasing Lys (*P* < 0.05). Based on broken-line and quadratic analysis models, dietary SID Lys levels for the minimum F:G were 0.94%. In Exp. 2, 650 finishing pigs (57.24 ± 2.00 kg bodyweight) were allotted to 5 dietary treatments providing SID Lys of 0.63, 0.71, 0.79, 0.87, and 0.95%. Each treatment had 5 replicates, 26 pigs per replication. The highest final bodyweight was achieved by 0.79% Lys while the highest average daily gain (ADG) and average daily feed intake (ADFI) was achieved by pigs consuming the 0.87% Lys diet (*P* < 0.05). Additionally, the lowest F:G was obtained by pigs fed the 0.79 and 0.87% Lys diet (*P* < 0.05). Based on broken-line and quadratic analysis models, the optimum Lys was 0.81 and 0.82% for ADG and F:G, respectively. In Exp. 3, 600 late finishing pigs (92.22 ± 2.41 kg bodyweight), were divided into 5 treatments providing Lys levels of 0.53, 0.60, 0.66, 0.73, and 0.79%. Each treatment had 5 replicates, 24 pigs per replication. Results showed that final bodyweight, ADG, ADFI, and F:G was not affected by increasing dietary Lys level, suggesting that the lowest SID Lys (0.53%) was sufficient for this group of pigs. Taken together, the SID Lys requirement for pigs from 30 to 60 kg, 60 to 90 kg, 90 to 120 kg was 0.94%, 0.81 to 0.82, and 0.53%, respectively, depending on the response criteria with performance maximized.

## Background

Numerous studies have shown that the nutritional requirements of pigs vary according to their age, gender, weight, breed, production potential, physiological state, and housing environment [1–3]. Recently, antibiotics as feed additives for growth promotion have been banned in China, which may require animal nutritionists and feed manufacturers to re-estimate the nutrient requirements, especially the dietary requirements of amino acids (AA) for maximal growth and optimal health when pigs are fed antibiotics-free diets. Dietary AA are essential for the survival, growth, development, reproduction and health of animals [4]. Feeding diets below the AA requirement may decrease body protein deposition and increase fat deposition.

Among AA, lysine (Lys) has been identified as first limiting for pigs fed corn-soybean meal diets [2, 5]. Lysine not only serves as a building block for proteins biosynthesis, but also participates in various physiological activities including regulation of other nutrient metabolism, modulation of the plasma AA profile, enhancing hormone production and immune functions [6–9]. Dietary supplementation of Lys has been shown to improve nitrogen retention and muscle protein accretion, as well as growth and production performance of pigs [10]. In contrast, Lys deficiency may decrease animal growth performance and elevate the susceptibility to infectious diseases [8, 11].

In commercial pig production, dietary Lys concentration has a big economic impact on feed cost. Under different economic scenarios, the biological Lys requirement to maximize growth rate may not maximize profitability. Therefore, to achieve ideal economic and environmental benefits, it is important to ensure appropriate levels of Lys are used in the diet [12]. The present study was designed to determine the optimal Lys concentrations for maximum growth performance and best feed efficiency for pigs with two different regression approaches. Given the fact that Lys requirements change with the different stages of production, three experiments were conducted to evaluate optimal Lys concentrations for growing (Exp. 1, 30 to 60 kg bodyweight), early finishing (Exp. 2, 60 to 90 kg), and late finishing (Exp. 3, 90 to 120 kg) pigs under commercial conditions.

## Materials and methods

### Animals and dietary treatments

The Hunan Normal University Animal Care and Use Committee reviewed and approved all animal protocols used in the current study (2019-170). Experiments were carried out at a commercial research facility (Twins Group Co., Ltd., Nanchang, China). The genotype of the pigs used in this study was Duroc × (Yorkshire × Landrace) hybrid. Pigs were housed in pens (7.0 × 5.0 m) equipped with slatted floors, a seven-hole self-feeder and a four-cup watering unit. Room temperature was maintained at approximately 22 to 24 °C and the humidity was controlled between 50 and 70%. Pigs were allowed ad libitum access to feed and water throughout the experiment.

In the current study, experimental diets were formulated to provide 80, 90, 100, 110, 120% of standardized ileal digestible (SID) Lys requirements estimated by NRC [13]. Diets in all experiments were formulated based on corn and soybean meal and Lys levels in diets were achieved by adjusting the amount of soybean meal and L-Lys (L-Lys sulfate 70%, Meihua Group, Hebei, China) using BESTMIX (ADIFO). All other nutrient requirements met the NRC [13] recommendations. All chemical analysis were conducted according the methods of the AOAC [14]. The nitrogen content of the diets was analyzed by KDN-103 automatic kjeldahl nitrogen analyzer (Huaye, Shanghai, China) and CP content was estimated as total nitrogen × 6.25. For the analysis of most AA, diets were hydrolyzed in 6 N HCl at 110 °C for 24 h. Total sulfur AA was measured after performic acid oxidation followed by acid hydrolysis, and tryptophan content was detected after alkaline hydrolysis. AA composition was analyzed using a L-8900 Amino Acid Analyzer (HITACHI, Tokyo, Japan). Three samples of each diet were analyzed. Ingredient, calculated and analyzed nutrient composition of the experimental diet formulations are shown in Tables 1, 2, and 3.

**Table 1:** Ingredient, calculated and analyzed nutrient composition of the diets for 30 to 60 kg growing pigs

Item	SID Lys, %				
	0.75	0.85	0.94	1.03	1.13
Ingredients, %					
Corn	80.42	78.45	76.67	73.89	69.88
Soybean meal (43.90% CP)	11.49	15.20	18.53	21.81	25.42
Wheat bran	4.15	2.33	0.70		
Soybean oil				0.17	0.53
Limestone	1.16	1.13	1.11	1.06	1.05
Mono-calcium and di-calcium phosphate	1.05	1.05	1.05	1.03	0.99
Salt	0.40	0.40	0.40	0.40	0.40
Choline chloride	0.07	0.07	0.07	0.07	0.07
Vitamin-mineral premix*	0.50	0.50	0.50	0.50	0.50
L-Lys sulfate 70.00%	0.55	0.59	0.62	0.66	0.69
L-Thr 98.50%	0.10	0.12	0.14	0.15	0.17
DL-Met 99.00%	0.03	0.06	0.10	0.13	0.16
L-Val 99.00%	0.04	0.06	0.08	0.10	0.12
L-Trp 98.00%	0.03	0.03	0.03	0.03	0.04
Total	100	100	100	100	100
Calculated composition					
CP, %	12.81	14.07	15.21	16.38	17.70
NE, Mcal/kg	2.48	2.48	2.48	2.48	2.48
SID-Lys/NE, g/Mcal	3.02	3.43	3.79	4.15	4.56
Ca, %	0.67	0.67	0.67	0.66	0.66
P, %	0.54	0.55	0.55	0.55	0.55
STTD-P, %	0.31	0.31	0.31	0.31	0.31
SID-Lys, %	0.75	0.85	0.94	1.03	1.13
SID-Met+Cys/Lys, %	57.16	57.00	57.00	57.00	57.00
SID-Met/Lys, %	29.00	31.23	32.95	34.03	34.78
SID-Thr/Lys, %	61.00	61.00	61.00	61.00	61.00
SID-Trp/Lys, %	18.00	18.00	18.00	18.00	18.00
SID-Val/Lys, %	66.00	66.00	66.00	66.00	66.00
SID-Ile/Lys, %	53.00	53.00	53.00	53.00	53.00
SID-Leu/Lys, %	137.99	131.43	126.72	122.38	118.00
SID-His/Lys, %	39.60	38.57	37.83	37.24	36.69
SID-Arg/Lys, %	83.05	83.76	84.28	84.86	85.51
SID-Phe/Lys, %	67.92	66.87	66.11	65.42	64.73
SID-Tyr/Lys, %	44.59	43.85	43.32	42.96	42.69
Analyzed values, %					
CP	13.52	14.64	15.56	16.68	17.92
EE	3.28	3.21	3.12	2.99	2.94
CF	2.87	2.95	2.98	3.11	3.24
CA	4.51	4.63	4.71	4.79	4.92
Lys	0.90	0.99	1.10	1.18	1.25
Met+Cys	0.54	0.59	0.65	0.71	0.77
Met	0.27	0.31	0.36	0.40	0.45
Thr	0.57	0.65	0.67	0.77	0.82
Trp	0.17	0.18	0.20	0.20	0.22
Val	0.62	0.69	0.75	0.83	0.89
Ile	0.57	0.62	0.68	0.74	0.79
Leu	1.23	1.33	1.42	1.51	1.58
His	0.38	0.41	0.44	0.47	0.51
Arg	0.73	0.82	0.91	0.99	1.09
Phe	0.62	0.69	0.75	0.81	0.87
Tyr	0.44	0.49	0.51	0.54	0.58

Abbreviations: *SID* standardized ileal digestible, *Lys* lysine, *CP* crude protein, *NE* net energy, *STTD* standardized total tract digestible, *Met* methionine, *Cys* cysteine, *Thr* threonine, *Trp* tryptophan, *Val* valine, *Ile* isoleucine, *Leu* leucine, *His* histidine, *Arg* arginine, *Phe* phenylalanine, *Tyr* tyrosine, *EE* ether extract, *CF* crude fiber, *CA* crude ash

*Premix provided the following per kilogram of complete diet: vitamin A, 9000 IU; vitamin D_3_, 2400 IU; vitamin E, 20 IU; vitamin K_3_, 3 mg; thiamine, 1.4 mg; riboflavin, 4 mg; pyridoxine, 3 mg; vitamin B12, 12 μg; nicotinic acid, 30 mg; pantothenic acid, 14 mg; folic acid, 0.8 mg; biotin, 44 μg; Fe, 76 mg; Cu, 240 mg; Zn, 76 mg; Mn, 20 mg; I, 0.48 mg; Se, 0.4 mg

**Table 2:** Ingredient, calculated and analyzed nutrient composition of the diets for 60 to 90 kg finishing pigs

Item	SID Lys, %				
	0.63	0.71	0.79	0.87	0.95
Ingredients, %					
Corn	82.30	80.74	79.16	77.58	76.00
Soybean meal (43.90% CP)	6.92	9.88	12.85	15.81	18.78
Wheat bran	7.27	5.81	4.36	2.90	1.45
Limestone	1.12	1.10	1.07	1.05	1.03
Mono-calcium and di-calcium phosphate	0.79	0.79	0.79	0.80	0.80
Salt	0.40	0.40	0.40	0.40	0.40
Choline chloride	0.07	0.07	0.07	0.07	0.07
Vitamin-mineral premix*	0.50	0.50	0.50	0.50	0.50
L-Lys sulfate 70.00%	0.51	0.54	0.57	0.60	0.63
L-Thr 98.50%	0.09	0.10	0.12	0.13	0.15
DL-Met 99.00%	0.01	0.02	0.04	0.07	0.10
L-Val 99.00%	0.01	0.03	0.04	0.06	0.08
L-Trp 98.00%	0.03	0.03	0.03	0.03	0.03
Total	100	100	100	100	100
Calculated composition					
CP, %	11.35	12.35	13.36	14.37	15.39
NE, Mcal/kg	2.48	2.48	2.48	2.48	2.48
SID-Lys/NE, g/Mcal	2.54	2.86	3.19	3.51	3.83
Ca, %	0.60	0.60	0.60	0.60	0.60
P, %	0.50	0.50	0.50	0.50	0.50
STTD-P, %	0.27	0.27	0.27	0.27	0.27
SID-Lys, %	0.63	0.71	0.79	0.87	0.95
SID-Met+Cys/Lys, %	60.65	57.85	57.00	57.00	57.00
SID-Met/Lys, %	28.00	28.00	29.38	31.20	32.71
SID-Thr/Lys, %	62.00	62.00	62.00	62.00	62.00
SID-Trp/Lys, %	18.00	18.00	18.00	18.00	18.00
SID-Val/Lys, %	66.00	66.00	66.00	66.00	66.00
SID-Ile/Lys, %	53.00	53.00	53.00	53.00	53.00
SID-Leu/Lys, %	148.23	140.81	134.88	130.04	126.01
SID-His/Lys, %	41.34	40.16	39.22	38.45	37.80
SID-Arg/Lys, %	82.08	82.87	83.51	84.03	84.46
SID-Phe/Lys, %	69.58	68.39	67.43	66.65	66.00
SID-Tyr/Lys, %	45.93	45.07	44.39	43.83	43.37
Analyzed values, %					
CP	11.93	12.84	13.74	14.63	15.52
EE	3.47	3.39	3.33	3.21	3.14
CF	2.86	2.95	2.97	3.04	3.05
CA	4.08	4.18	4.28	4.36	4.45
Lys	0.74	0.83	0.90	0.95	1.08
Met+Cys	0.50	0.52	0.54	0.60	0.67
Met	0.23	0.24	0.28	0.32	0.37
Thr	0.50	0.56	0.60	0.63	0.68
Trp	0.14	0.15	0.17	0.19	0.19
Val	0.54	0.59	0.64	0.71	0.77
Ile	0.51	0.55	0.58	0.64	0.70
Leu	1.12	1.21	1.26	1.37	1.43
His	0.32	0.37	0.40	0.42	0.43
Arg	0.61	0.70	0.76	0.83	0.90
Phe	0.52	0.60	0.66	0.72	0.79
Tyr	0.39	0.43	0.44	0.50	0.50

Abbreviations: *SID* standardized ileal digestible, *Lys* lysine, *CP* crude protein, *NE* net energy, *STTD* standardized total tract digestible, *Met* methionine, *Cys* cysteine, *Thr* threonine, *Trp* tryptophan, *Val* valine, *Ile* isoleucine, *Leu* leucine, *His* histidine, *Arg* arginine, *Phe* phenylalanine, *Tyr* tyrosine, *EE* ether extract, *CF* crude fiber, *CA* crude ash

*Premix provided the following per kilogram of complete diet: vitamin A, 9000 IU; vitamin D_3_, 2400 IU; vitamin E, 20 IU; vitamin K_3_, 3 mg; thiamine, 1.4 mg; riboflavin, 4 mg; pyridoxine, 3 mg; vitamin B12, 12 μg; nicotinic acid, 30 mg; pantothenic acid, 14 mg; folic acid, 0.8 mg; biotin, 44 μg; Fe, 76 mg; Cu, 240 mg; Zn, 76 mg; Mn, 20 mg; I, 0.48 mg; Se, 0.4 mg

**Table 3:** Ingredient, calculated and analyzed nutrient composition of the diets for 90 to 120 kg late-finishing pigs

Item	SID Lys, %				
	0.53	0.60	0.66	0.73	0.79
Ingredients, %					
Corn	83.57	82.22	81.04	79.65	78.45
Soybean meal (43.90% CP)	3.46	6.10	8.36	11.00	13.27
Wheat bran	9.85	8.53	7.41	6.11	5.00
Limestone	1.02	1.00	0.98	0.96	0.94
Mono-calcium and di-calcium phosphate	0.59	0.59	0.59	0.59	0.59
Salt	0.40	0.40	0.40	0.40	0.40
Choline chloride	0.07	0.07	0.07	0.07	0.07
Vitamin-mineral premix	0.50	0.50	0.50	0.50	0.50
L-Lys sulfate 70.00%	0.46	0.48	0.50	0.53	0.55
L-Thr 98.50%	0.07	0.08	0.09	0.11	0.12
DL-Met 99.00%		0.01	0.01	0.02	0.04
L-Val 99.00%			0.01	0.02	0.04
L-Trp 98.00%	0.02	0.02	0.02	0.03	0.03
Total	100	100	100	100	100
Calculated composition					
CP, %	10.26	11.14	11.90	12.79	13.56
NE, Mcal/kg	2.48	2.48	2.48	2.48	2.48
SID-Lys/NE, g/Mcal	2.14	2.42	2.66	2.94	3.19
Ca, %	0.52	0.52	0.52	0.52	0.52
STTD-P, %	0.24	0.24	0.24	0.24	0.24
SID-Lys, %	0.53	0.60	0.66	0.73	0.79
SID-Met+Cys/Lys, %	68.19	63.67	61.24	58.92	58.00
SID-Met/Lys, %	29.98	29.00	29.00	29.00	29.75
SID-Thr/Lys, %	63.00	63.00	63.00	63.00	63.00
SID-Trp/Lys, %	18.00	18.00	18.00	18.00	18.00
SID-Val/Lys, %	69.39	66.58	66.00	66.00	66.00
SID-Ile/Lys, %	54.00	54.00	54.00	54.00	54.00
SID-Leu/Lys, %	161.78	152.73	146.48	140.48	136.18
SID-His/Lys, %	43.98	42.52	41.51	40.55	39.86
SID-Arg/Lys, %	82.66	83.61	84.26	84.89	85.34
SID-Phe/Lys, %	72.65	71.19	70.18	69.21	68.51
SID-Tyr/Lys, %	48.26	47.18	46.44	45.74	45.23
Analyzed values, %					
CP	10.02	11.35	12.01	12.63	13.51
EE	3.59	3.78	3.46	3.41	3.31
CF	2.81	2.89	2.91	2.96	3.01
CA	3.72	3.78	3.87	3.96	4.02
Lys	0.64	0.68	0.78	0.82	0.90
Met+Cys	0.45	0.50	0.51	0.54	0.56
Met	0.20	0.23	0.23	0.26	0.27
Thr	0.45	0.46	0.54	0.59	0.62
Trp	0.12	0.14	0.15	0.16	0.18
Val	0.47	0.49	0.55	0.61	0.63
Ile	0.46	0.47	0.53	0.57	0.57
Leu	1.03	1.11	1.14	1.22	1.23
His	0.31	0.31	0.35	0.36	0.39
Arg	0.53	0.58	0.67	0.70	0.77
Phe	0.47	0.51	0.56	0.60	0.62
Tyr	0.36	0.40	0.40	0.43	0.47

Abbreviations: *SID* standardized ileal digestible, *Lys* lysine, *CP* crude protein, *NE* net energy, *STTD* standardized total tract digestible, *Met* methionine, *Cys* cysteine, *Thr* threonine, *Trp* tryptophan, *Val* valine, *Ile* isoleucine, *Leu* leucine, *His* histidine, *Arg* arginine, *Phe* phenylalanine, *Tyr* tyrosine, *EE* ether extract, *CF* crude fiber, *CA* crude ash

*Premix provided the following per kilogram of complete diet: vitamin A, 6000 IU; vitamin D_3_, 2400 IU; vitamin E, 20 IU; vitamin K_3_, 2 mg; thiamine, 0.96 mg; riboflavin, 5.3 mg; pyridoxine, 2 mg; vitamin B12, 12 μg; nicotinic acid, 22 mg; pantothenic acid, 11.2 mg; folic acid, 0.4 mg; biotin, 40 μg; Fe, 76 mg; Cu, 120 mg; Zn, 76 mg; Mn, 12 mg; I, 0.24 mg; Se, 0.4 mg

In Exp. 1, a total of 650 growing pigs, weighing initially 32.21 ± 0.33 kg, were allocated randomly to 5 dietary treatments. SID Lys levels were designed at 0.75, 0.85, 0.94, 1.03, and 1.13%. Each treatment had 5 replicate pens with 26 pigs per pen with a similar number of barrows and gilts in each pen. Experiment was conducted for 30 days. In Exp. 2, 650 finishing pigs (57.24 ± 2.00 kg bodyweight) were randomly allotted to 5 dietary treatments providing SID Lys levels of 0.63, 0.71, 0.79, 0.87, and 0.95%. Each treatment had 5 replicates per treatment, 26 pigs per replication. Experiment lasted for 35 days. In Exp. 3, 600 late finishing pigs, weighing an average of 92.22 ± 2.41 kg, were used in a 30-day growth trial. Pigs were randomly divided into 5 dietary treatments with 5 replicates per treatment, and 24 pigs per replication. Experimental diets were formulated to contain 5 SID Lys concentrations (0.53, 0.60, 0.66, 0.73, and 0.79%). The ideal ratios of other AA to Lys were kept similar among the diets.

### Data collection and statistical analysis

Pigs per pen were weighed at trial initiation and termination and feed disappearance was measured to determine average daily gain (ADG), average daily feed intake (ADFI), and feed to gain ratio (F:G). Pigs were monitored daily throughout the experimental period. Diarrhea rate was calculated as follows: Diarrhea rate = ([number of pigs with diarrhea × number of days of diarrhea]/[total number of pigs × number of days of experiment]) × 100%.

Data were checked for normality by D’Agostino & Pearson normality test and diarrhea rate was analyzed by Kruskal-Wallis test. Statistical differences were determined using one-way ANOVA followed by Tukey’s post hoc analysis. Polynomial contrasts were performed to determine linear and quadratic relationships. Significance was defined as a *P*-value < 0.05, while 0.05 < *P* < 0.1 was used to indicate a tendency towards significance. Estimates of Lys requirements for optimum performance were determined by subjecting the ADG and F:G pen average data to the linear broken-line [y = L + U × (R − x), where (R − x) is zero when x > R], and quadratic [y = L + U × (R − x)^2^, where (R − x) is zero when x > R] regression models as described by Robbins et al. [15], using the NLIN procedure of SAS (SAS Inst. Inc., Cary, NC).

## Results

### Experiment 1

As shown in Table 4, there was significant difference in ADFI among groups. The lowest F:G was obtained by pigs fed the 1.03% SID Lys diet while the highest F:G was obtained at 0.75 and 1.13% (Table 4). Moreover, F:G showed both linear (*P* < 0.05) and quadratic (*P* < 0.05) reduction with increasing SID Lys concentration in the diet. Daily Lys intake and Lys efficiency for bodyweight increased linearly (*P* < 0.05) with increasing dietary Lys level while Lys efficiency also increased in a quadratic manner. However, the effect of dietary SID Lys was not observed for final bodyweight, ADG, and diarrhea rate in Exp. 1.

**Table 4:** Effect of dietary SID Lys level on the performance of pigs from 30 to 60 kg (Exp.1)

Item	SID Lys, %					SEM	*P* value		
	0.75	0.85	0.94	1.03	1.13		ANOVA	Linear	Quadratic
Initial bodyweight, kg	32.25	32.21	32.23	32.17	32.21	0.07	1.00	0.80	0.92
Final bodyweight, kg	56.03	57.27	57.81	56.75	56.96	0.38	0.70	0.64	0.28
Average daily gain, kg	0.79	0.84	0.85	0.82	0.83	0.01	0.67	0.62	0.26
Average daily feed intake, kg	1.76	1.74	1.75	1.62	1.73	0.02	0.05	0.11	0.36
Feed to gain ratio	2.24^a^	2.09^ab^	2.06^ab^	1.98^b^	2.10^a^	0.03	0.02	0.02	0.01
Daily SID Lys intake, g	13.20	14.74	16.48	16.69	19.55	0.45	0	0	0.27
Lys efficiency for bodyweight	16.78^d^	17.75^cd^	19.34^bc^	20.37^b^	23.69^a^	0.52	0	0	0.01
^#^Diarrhea rate, %	0.03	0	0	0	0.05	*P* = 0.21			

Abbreviations: *SID* standardized ileal digestible, *Lys* lysine, *n* = 5, *SEM* standard error of mean, Lys efficiency for bodyweight = Daily SID Lys intake (g)/ Average daily gain (kg)

^#^Diarrhea rate was analyzed by Kruskal-Wallis test

**Fig. 1:**
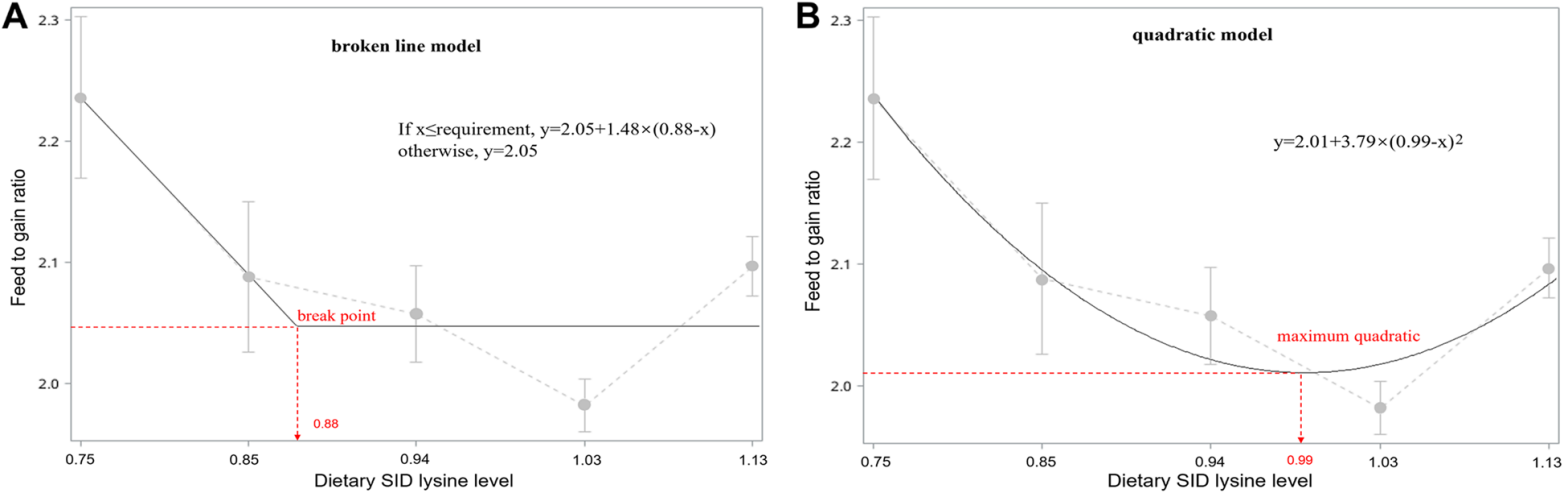
Estimation of SID Lys for 30 to 60 kg growing pigs (Exp. 1). Data points represent treatment means. Fitted broken-line (**a**) and quadratic (**b**) plot of F:G as a function. The optimal Lys requirement determined by broken-line analysis was 0.88 (y plateau = 2.05, *R*^2^ = 0.80), and by quadratic analysis was 0.99 (y plateau = 2.01, *R*^2^ = 0.92)

In this experiment, F:G was used for regression analysis, as it showed both linear and quadratic response with an increasing level of dietary SID Lys. Broken-line models described the dietary SID Lys levels for the minimum F:G was 0.88% (Fig. 1A). Moreover, based on quadratic models, the optimum Lys levels to minimize F:G for 30 to 60 kg growing pigs were 0.99% (Fig. 1B). The average SID Lys requirements estimated by the two models was 0.94%.

### Experiment 2

The highest final bodyweight was achieved by pigs consuming diets containing 0.79% SID Lys while the lowest final bodyweight was obtained at 0.63% (*P* < 0.05) (Table 5). Moreover, with increasing Lys supply, final bodyweight showed a quadratic increase (*P* < 0.05) and tended to increase linearly (*P* = 0.051). The highest ADG and ADFI was achieved by pigs fed the 0.87% SID Lys diet whereas the lowest ADG and ADFI was obtained at 0.63%. In addition, ADG increased linearly (*P* < 0.05) and quadratically (*P* < 0.05) as SID Lys increased, while ADFI increased in a quadratic manner (*P* < 0.05) and a tendency to increase (*P* = 0.074) with increasing Lys content in the diet. There was a linear (*P* < 0.05) and quadratic (*P* < 0.05) improvement in F:G as SID Lys increased, and the lowest F:G was obtained by pigs fed the 0.79 and 0.87% SID Lys diet. Moreover, daily Lys intake and Lys efficiency for bodyweight increased linearly (*P* < 0.05) and quadratically (*P* < 0.05) with an increasing level of the SID Lys. No diarrhea was observed during the experiment period.

**Table 5:** Effect of dietary SID Lys level on the performance of pigs from 60 to 90 kg (Exp.2)

Item	SID Lys, %					SEM	*P* value		
	0.63	0.71	0.79	0.87	0.95		ANOVA	Linear	Quadratic
Initial bodyweight, kg	57.25	57.85	57.32	56.05	57.73	0.40	0.67	0.77	0.73
Final bodyweight, kg	88.29^b^	90.49^ab^	93.17^a^	92.58^ab^	90.94^ab^	0.58	0.05	0.05	0.02
Average daily gain, kg	0.89^c^	0.93^bc^	1.02^ab^	1.04^a^	0.95^bc^	0.02	0	0	0
Average daily feed intake, kg	2.40^b^	2.54^ab^	2.52^ab^	2.58^a^	2.48^ab^	0.02	0.02	0.07	0.01
Feed to gain ratio	2.71	2.72	2.47	2.47	2.62	0.03	0.02	0.04	0.05
Daily SID Lys intake, g	15.13	18.00	19.94	22.41	23.58	0.63	0	0	0.01
Lys efficiency for bodyweight	17.08^d^	19.32^c^	19.54^bc^	21.52^b^	24.88^a^	0.57	0	0	0.04
Diarrhea rate, %	0	0	0	0	0				

Abbreviations: *SID* standardized ileal digestible, *Lys* lysine, *n* = 5, *SEM* standard error of mean, Lys efficiency for bodyweight = Daily SID Lys intake (g)/ Average daily gain (kg)

**Fig. 2:**
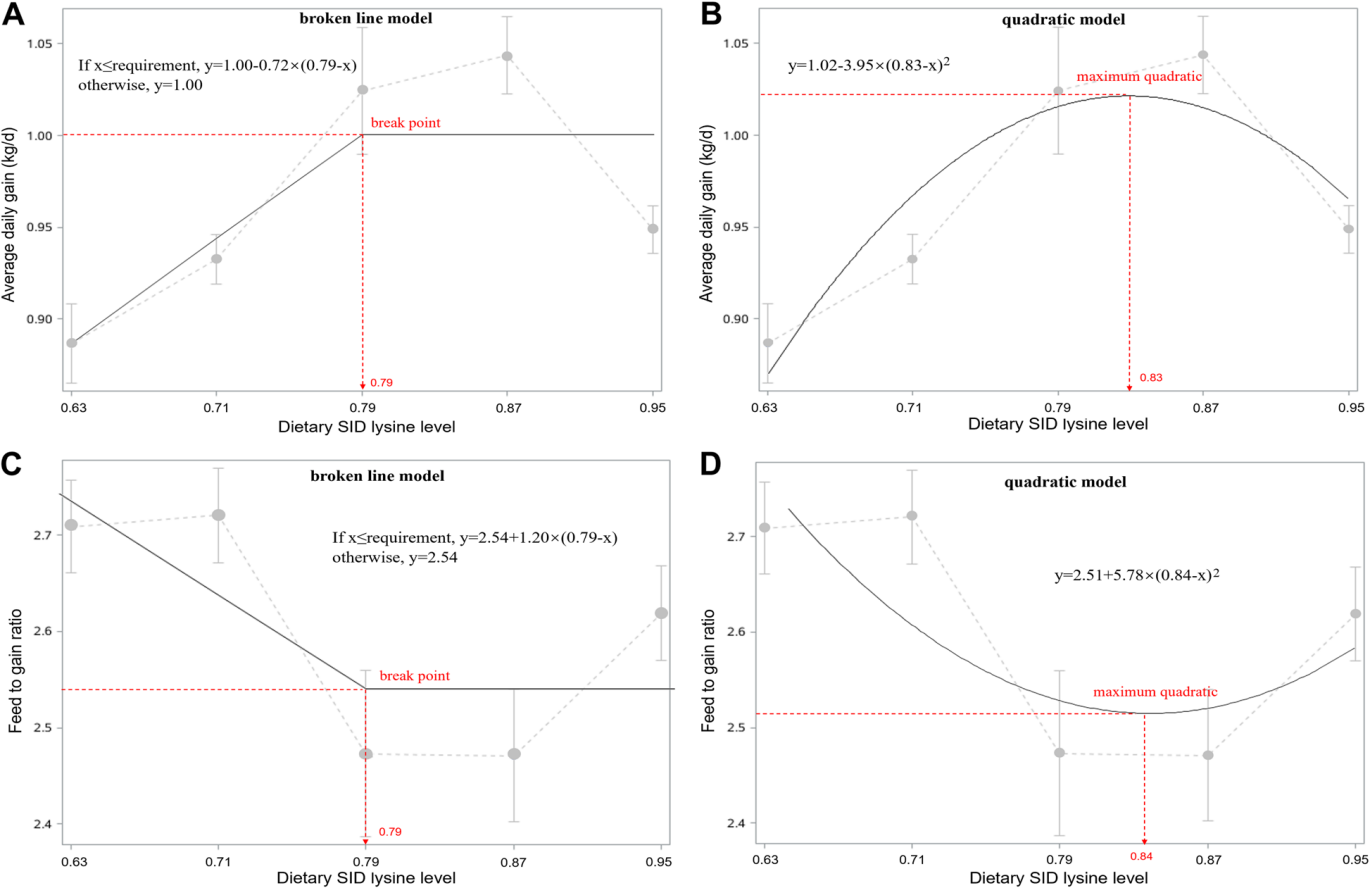
Estimation of SID Lys for 60 to 90 kg finishing pigs (Exp. 2). Data points represent treatment means. The optimal Lys requirement determined by broken-line analysis was 0.79 (y plateau = 1.00, *R*^2^ = 0.70), and by quadratic analysis was 0.83 (y plateau = 1.02, *R*^2^ = 0.85). Fitted broken-line (**c**) and quadratic (**d**) plot of F:G as a function. The optimal Lys requirement determined by broken-line analysis was 0.79 (y plateau = 2.54, *R*^2^ = 0.61), and by quadratic analysis was 0.84 (y plateau = 2.51, *R*^2^ = 0.63)

Using broken-line models, the breakpoint for ADG and F:G of 60 to 90 kg finishing pigs both occurred at 0.79% SID Lys (Fig. 2A and C). In addition, the quadratic analysis models estimated the optimum SID Lys as 0.83 and 0.84% to maximize ADG and minimize F:G, respectively (Fig. 2B and D). The average SID Lys requirements estimated by the two models was 0.81 and 0.82%, using ADG and F:G as the response criteria, respectively.

### Experiment 3

Daily Lys intake and Lys efficiency for bodyweight increased linearly (*P* < 0.05) with increasing dietary Lys level (Table 6). However, final bodyweight, ADG, ADFI, and F:G was not affected by increasing dietary Lys level. No diarrhea was observed during the experiment period. In this experiment, due to lack of significant difference, no data were subjected to regression analysis.

**Table 6:** Effect of dietary SID Lys level on the performance of pigs from 90 to 120 kg (Exp.3)

Item	SID Lys, %					SEM	*P* value		
	0.53	0.60	0.66	0.73	0.79		ANOVA	Linear	Quadratic
Initial bodyweight, kg	91.49	92.61	92.07	92.80	92.12	0.48	0.94	0.69	0.59
Final bodyweight, kg	118.76	120.55	120.46	120.23	119.87	0.45	0.76	0.57	0.27
Average daily gain, kg	0.91	0.93	0.95	0.91	0.93	0.01	0.92	0.87	0.55
Average daily feed intake, kg	2.79	2.83	2.83	2.81	2.84	0.02	0.90	0.58	0.81
Feed to gain ratio	3.09	3.07	2.99	3.07	3.04	0.04	0.95	0.73	0.72
Daily SID Lys intake, g	14.79	17.00	18.65	20.48	22.41	0.55	0	0	0.90
Lys efficiency for bodyweight	16.37^c^	18.42^bc^	19.73^b^	22.41^a^	24.25^a^	0.62	0	0	0.69
Diarrhea rate, %	0	0	0	0	0				

Abbreviations: *SID* standardized ileal digestible, *Lys* lysine, *n* = 5, *SEM* standard error of mean, Lys efficiency for bodyweight = Daily SID Lys intake (g)/ Average daily gain (kg)

## Discussion

Given that the appropriate SID Lys requirement plays an important role in reducing feed costs with maintaining animal performance, this study was conducted to determine the optimal SID Lys requirements of growing-finishing pigs under commercial conditions. To avoid the possible compensatory growth effect [16, 17], the pigs used in the three experiments demonstrated herein were obtained from three different batches.

In Exp. 1 and 2, growth performance was markedly affected by the SID Lys supply. For 30 to 60 kg growing pigs in Exp. 1, there was significant difference in ADFI among groups. The impact of different SID Lys levels on feed intake of ad libitum fed pigs was not consistent in the literature. Several studies revealed that Lys supply did not affect the feed intake [18], while other reports showed that Lys restriction resulted in reduced [19] or elevated feed intake [20]. This difference appears to be partly due to the different method used to formulate Lys-restricted feeds, by varying proportions of feed ingredients or using synthetic AA to create restriction [1]. Moreover, dietary fiber (wheat bran) or soy oil used in the experimental diets may also affect feed intake. According to Yin et al. [20], gut microbiome may also contribute to the potential mechanism of lysine restriction-mediated feeding behavior.

In Exp.1, the lowest F:G was obtained by pigs fed the 1.03% SID Lys diet while the highest F:G was obtained at 0.75 and 1.13% SID Lys. Notably, F:G showed both linear and quadratic reduction with increasing SID Lys inclusion. Our results were in line with the results by [1], where the authors observed that, for 25 to 50 kg pigs, lower G:F ratio was observed in pigs fed the reduced SID Lys diet. However, in terms of ADG, these authors reported that ADG significantly increased as SID Lys increased while no difference in ADG for growing pigs was found in our study.

For early finishing pigs from 60 to 90 kg in Exp. 2, maximum ADG and ADFI were achieved by pigs consuming the 0.87% SID Lys diet. In addition, ADG increased linearly and quadratically as SID Lys increased, while ADFI increased in a quadratic manner and a tendency to increase with increasing SID Lys in the diet. There was a linear and quadratic improvement in F:G as SID Lys increased, and the lowest F:G was obtained by pigs fed the 0.79 and 0.87% Lys diet. These results were in accordance with previous studies [10, 18], who showed that Lys restriction had significantly influence on growth performance traits such as ADG, ADFI and G:F ratio. Moreover, data compiled by Cloutier et al. [1] also revealed that ADG for finishing pigs (70 to 100 kg) significantly increased with increasing Lys levels while no difference was observed with respect to ADFI and G:F ratios.

For 90 to 120 kg late finishing pigs in Exp. 3, there was no evidence for difference in terms of ADG, ADFI, and F:G, suggesting that the performance of pigs from 90 to 120 kg was less affected by SID Lys restriction and the lowest SID Lys (0.53%) seemed to be sufficient for this group of pigs. A previous study carried out by Ma et al., [21] showed that increasing SID Lys could improve ADG and FCR (feed conversion ratio) both in linear and quadratic manner for late finishing gilts fed low crude protein. Another research also found that increasing SID Lys increased ADG and ADFI quadratically in finishing pigs weighing greater than 100 kg, while marginal improvements in F:G were observed with increasing SID Lys [12]. The inconsistency from the present study could be due to differences in Lys and crude protein levels, animal gender, weight, as well as experimental conditions.

In the current study, the appropriate Lys requirement was estimated with a linear-break point model and a quadratic model using ADG and F:G as the response criteria. Noteworthy, we noticed that different statistical models could yield different requirement estimates, in accordance with previous studies [22–24]. Based on broken-line models, the dietary SID Lys levels for the minimum F:G was 0.88%, lower than the NRC [13] recommendations of 0.94% for 30 to 60 kg growing pigs. Breakpoint for ADG and F:G of 60 to 90 kg finishing pigs both occurred at 0.79% SID Lys, which was the same as the current NRC recommendations. In contrast, when using a quadratic model, the optimum SID Lys levels to minimize F:G of 30 to 60 kg pigs were 0.99%, slightly higher than the current NRC [13] recommendations. A previous study carried out by Ho et al. [25] showed that the optimal SID Lys requirement for 30-50 kg pigs was 1.10%, which was also higher than the NRC [13] recommendations. Consistently, for 60 to 90 kg pigs, estimation of the required Lys for ADG and F:G was 0.83 and 0.84% using quadratic regression, which was greater than the NRC recommendations.

Interestingly, we found that F:G resulted in higher optimum SID Lys requirement than ADG in this study. Similar observations where F:G gave higher estimates of Lys requirement compared to ADG have been reported previously in the literature [2, 26]. Hence, these results may indicate that the Lys requirement would differ depending on the response criteria. More nutrients would be partitioned towards maintenance requirement when bodyweight increase was more towards visceral organs [2]. Therefore, even as maximal growth was attained, there could still be a metabolic demand for Lys [27]. Furthermore, in the present study, we found that the estimated Lys requirements were notably lower using broken-line models than quadratic models. In accordance with this, previous work also observed that broken-line models always resulted in lower estimates compared with quadratic models [28, 29]. As mentioned in previous studies [21, 30], the Lys requirement was often underestimated using broken-line regression model, because the breakpoint was selected as the minimum nutrient requirement for the theoretical average pig. However, on the contrary, the quadratic model was used to estimate the Lys requirement to reach 100% of the maximum response, which usually appeared to overestimate the nutritional requirement for pigs [30, 31]. For quadratic model, the arbitrary selection of 90% or 95% of the maximum response was probably aimed at meeting the requirement of most of the animals in a population [32]. Therefore, taking the average of the two models would be more closer to the requirement of the pigs or economic optimum.

Taken together, the SID Lys requirement for pigs from 30 to 60 kg bodyweight was 0.94%, for pigs from 60 to 90 kg was 0.81 to 0.82%, and for pigs from 90 to 120 kg was 0.53% depending on the response criteria with performance maximized. Using the estimated growth performance equations provided may aid swine nutritionists to determine the most economical Lys levels in actual diet formulation for a given situation.
